# *Pinellia pedatisecta* agglutinin-based lectin blot analysis distinguishes between glycosylation patterns in various cancer cell lines

**DOI:** 10.3892/ol.2014.2201

**Published:** 2014-05-30

**Authors:** NA LI, GUOPING DONG, SHUANGHUI WANG, SHIPING ZHU, YI SHEN, GONGCHU LI

**Affiliations:** College of Life Sciences, Zhejiang Sci-Tech University, Hangzhou, Zhejiang 310018, P.R. China

**Keywords:** *Pinellia pedatisecta* agglutinin, lectin blot, glycosylation fingerprint

## Abstract

The analysis of altered glycosylation patterns may provide biomarkers for various types of cancer. The present study developed a *Pinellia pedatisecta* agglutinin (PPA)-based lectin blot analysis technique, which was used to analyze the glycosylation patterns in various types of cancer cells. Results showed that a typical band located between 47 and 85 kDa was obtained in the HL60 leukemia cells, whereas three typical bands located between 20 and 47 kDa were observed in the Kasumi-1 leukemia cells. For the PLC, BEL-7404, Huh7 and H1299 solid tumor cell lines, different band patterns were detected, with bands typically located between 55 and 100 kDa. The findings of the present study show that PPA-based lectin blot analysis is capable of distinguishing between glycosylation patterns in leukemia and solid tumor cell lines. The glycofiles detected using PPA-based lectin blot analysis may provide a ‘glycosylation fingerprint’ for a variety of cancer cells, which may be valuable for cancer prognosis and diagnosis.

## Introduction

Altered glycosylation has been reported in various types of cancer and may have a role in cancer metastasis and progression (1–3). Lectins are carbohydrate-binding proteins that contain at least one non-catalytic domain that binds reversibly with mono- or oligosaccharides with high specificity (4). Therefore, lectins may be useful tools for analyzing glycofiles and may be used as biomarkers for a variety of types of cancer, including aggressive breast (5,6), ovarian (7), pancreatic (8), prostate (9) and liver (10) cancer. The monocot mannose-binding lectin, *Pinellia pedatisecta* agglutinin (PPA), accumulates in the tuber of *P. pedatisecta*, a species of the Araceae family. In our previous studies, recombinant PPA was used in labeling fractions of myeloid leukemia cells (11) and was found to preferentially recognize drug-resistant cancer cells (12). The binding of drug-resistant K562/ADR leukemia cells with PPA enhanced the macrophage-induced phagocytosis of the K562/ADR cells, and the target of PPA on the K562/ADR cells was determined to be sarcolemmal membrane-associated protein (12). Furthermore, the exogenous expression of PPA using gene delivery has been found to induce cancer cell death through interacting with the methylosome, which contains methylosome protein 50 and protein arginine methyltransferase 5 (13). These studies indicate that PPA may be further developed to analyze glycosylation profiles in different types of cancer.

Lectin blot analysis is a biochemical technique that is similar to western blot analysis, in which tagged lectins are used as probes to detect glycosylation profiles in biological samples (14), including cancer plasma (15), as well as sera and tissue samples (16). In the present study, a novel PPA-based lectin blot analysis technique was developed using soluble Coxsackie-adenovirus receptor-PPA domain b fusion protein (sCAR-PPAb) as a probe. PPA-based lectin blot analysis detected typical glycofiles for various cancer cell lines, including leukemia and solid tumor cell lines.

## Materials and methods

### Cells

All cell lines were obtained from the American Type Culture Collection (Rockville, MD, USA). The HL-60 and Kasumi-1 human acute myeloid leukemia cell lines were maintained in RPMI-1640 medium (Hyclone Laboratories, Logan, UT, USA) supplemented with 10% fetal bovine serum (Life Technologies, Inc., Grand Island, NY, USA) and 1% L-glutamine (Life Technologies, Inc.). The PLC, BEL-7404 and Huh7 human liver cancer cell lines and the H1299 human lung cancer cell line were maintained in Dulbecco’s modified Eagle’s medium (Hyclone Laboratories) supplemented with 10% fetal bovine serum and 1% L-glutamine.

### Production and purification of sCAR-PPAb protein

The construction of the pQE30-sCAR-PPA plasmid has been reported previously (11). In the present study, a PPA domain b fragment (D^146^-S^250^) was used to replace the PPA full-length fragment. The pQE30-sCAR-PPAb plasmid was transformed into *Escherichia coli* strain M15, and the expression of sCAR-PPAb was induced using isopropyl β-D-1-thiogalactopyranoside. Inclusion bodies were subjected to protein purification using a wash method. In brief, inclusion bodies were washed three times with 4 ml wash buffer containing 1.46 mg/ml EDTA, 0.01 M Tris-HCl (pH 8.0), 8 M urea and 0.7% β-mercaptoethanol (all Sigma-Aldrich, St. Louis, MO, USA) for 12 h each. Following each wash, supernatants were collected using centrifugation at 15,984 rpm for 15 min and analyzed using SDS-PAGE followed by Coomassie Brilliant Blue (Sigma-Aldrich) staining. The purified proteins were then renatured through dialyzing against phosphate-buffered saline at 4°C overnight. The production of sCAR-PPAb was then assessed using western blot analysis with a mouse anti-6 histidine (6his) monoclonal antibody (Santa Cruz Biotechnology, Inc., Santa Cruz, CA, USA) and the IRDye^®^ 800 donkey anti-mouse immunoglobulin (Ig) G secondary antibody (Li-Cor, Inc., Lincoln, NA, USA). Bands were analyzed using an Odyssey^®^ Infrared Imaging System (Li-Cor, Inc.).

### PPA-based lectin blot analysis

Cell lysates were subjected to SDS-PAGE and electroblotted onto nitrocellulose membranes. The membranes were then blocked using Tris-buffered saline-Tween 20 (TBS-T) containing 5% bovine serum albumin at room temperature for 2 h, followed by incubation with 1.5 μg/ml sCAR-PPAb at 4°C overnight. Membranes incubated without sCAR-PPAb were used as the controls. The membranes were then washed with TBS-T three times and incubated with a mouse anti-6his monoclonal antibody (Santa Cruz Biotechnology, Inc.) at 4°C overnight. The membranes were subsequently washed and incubated with IRDye 800 donkey anti-mouse IgG (Li-Cor, Inc.) secondary antibody for 1 h at room temperature followed by analysis using an Odyssey Infrared Imaging System (Li-Cor, Inc.).

## Results

### Production and purification of the sCAR-PPAb fusion protein

**Figure 1:**
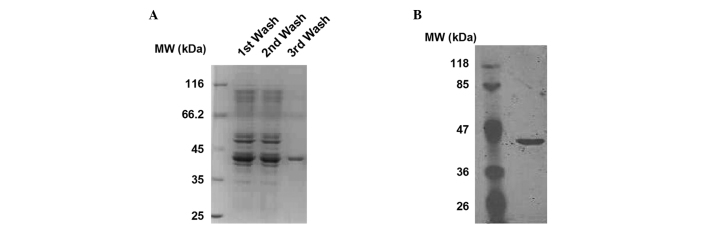
Production and purification of sCAR-PPAb. (A). Purification of sCAR-PPAb. The sCAR-PPAb protein was expressed as inclusion bodies in *E. Coli* M15 and purified using a three-step wash method. Inclusion bodies were washed three times using a wash buffer followed by centrifugation. The supernatants were collected and subjected to SDS-PAGE and the bands were visualized using Coomassie Brilliant Blue staining. (B). Western blot analysis of the 6 histidine (6his)-tag. Purified protein and a pre-stained protein molecular weight marker were subjected to SDS-PAGE and electroblotted onto a nitrocellulose membrane. The membrane was incubated with mouse anti-6his monoclonal antibodies followed by incubation with an IRDye^®^ 800 donkey anti-mouse immunoglobulin G secondary antibody. The bands were visualized using an Odyssey^®^ Infrared Imaging System. sCAR-PPAb, soluble coxsackie-adenovirus receptor-*Pinellia pedatisecta* agglutinin domain b; MW, molecular weight.

The sCAR-PPAb protein contains a 6his-tag, a human sCAR, a short flexible linker and a PPA domain b. In the present study, a bacterial expression system was used to produce sCAR-PPAb. The recombinant fusion protein was expressed as inclusion bodies in *E. coli* M15 and purified using a three-step wash method. Purification was verified using SDS-PAGE followed by Coomassie Brilliant Blue staining. As shown in Fig. 1A, a relatively pure protein, with a molecular weight of ~42 kDa, was obtained subsequent to the third wash. To further confirm the identity of the protein, western blot analysis was performed using an anti-6his antibody. As shown in Fig. 1B, the presence of the 6his-tag was verified. These findings show that the sCAR-PPAb fusion protein was successfully expressed and purified.

### PPA-based lectin blot analysis distinguishes typical glycofiles in various types of cancer cell

**Figure 2:**
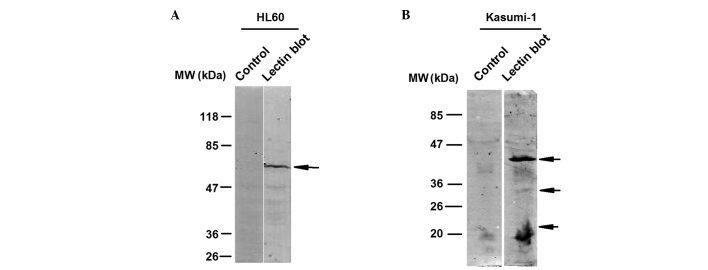
Glycosylation patterns of leukemia cells analyzed using PPA-based lectin blot analysis. (A). PPA-based lectin blot analysis of HL60 cells. Whole cell lysates of HL60 cells were subjected to SDS-PAGE and transferred onto nitrocellulose membranes. The membranes were then incubated with or without 1.5 μg/ml sCAR-PPAb overnight. The membrane incubated without sCAR-PPAb was used as the control. Following one wash, membranes were incubated with a mouse anti-6 histidine (6his) monoclonal antibody, and then an IRDye 800 donkey anti-mouse immunoglobulin G secondary antibody. The bands were visualized using an Odyssey^®^ Infrared Imaging System. (B) PPA-based lectin blot analysis of Kasumi-1 cells. Whole cell lysates of Kasumi-1 cells were subjected to SDS-PAGE and electroblotted onto nitrocellulose membranes. The membranes were then treated in the same way as those for the HL60 cells. sCAR-PPAb, soluble coxsackie-adenovirus receptor-*Pinellia pedatisecta* agglutinin domain b; MW, molecular weight.

A lectin blot was generated using sCAR-PPAb as the primary probe to detect the glycosylation pattern for a variety of cancer cells, including leukemia and solid tumor cell lines. As shown in Fig. 2A, the HL60 leukemia cell line was subjected to PPA-based lectin blot analysis and a typical band with a molecular weight of between 47 and 85 kDa was detected. Kasumi-1 leukemia cells were also subjected to PPA-based lectin blot analysis, in order to analyze whether other leukemia cells exhibited different detection patterns. Lectin blot analysis revealed three typical bands with various molecular weights in the Kasumi-1 cells (Fig. 2B), which were different to those of the HL60 cells, indicating that PPA-based lectin blot analysis is capable of distinguishing between glycosylation patterns in different leukemia cell lines.

**Figure 3:**
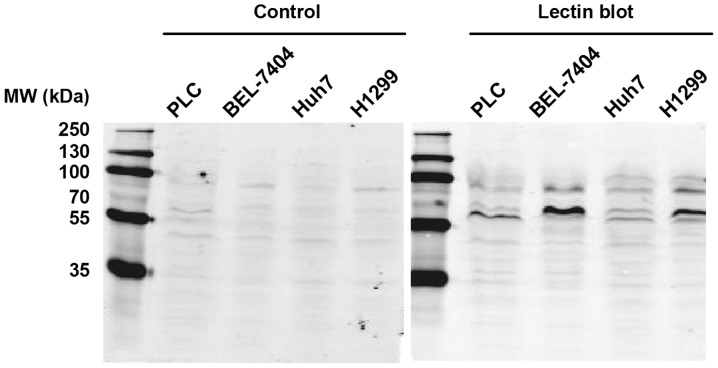
Glycosylation patterns of solid tumor cells analyzed using PPA-based lectin blot analysis. Whole cell lysates of PLC, BEL-7404, Huh7 and H1299 cells were subjected to SDS-PAGE and transferred onto nitrocellulose membranes. The membranes were then incubated with or without 1.5 μg/ml sCAR-PPAb overnight, followed by incubation with a mouse anti-6 histidine (6his) monoclonal antibody and an IRDye^®^ 800 donkey anti-mouse immunoglobulin G secondary antibody. A membrane treated without sCAR-PPAb was used as the control. The bands were visualized using an Odyssey^®^ Infrared Imaging System. sCAR-PPAb, soluble coxsackie-adenovirus receptor-*Pinellia pedatisecta* agglutinin domain b; MW, molecular weight.

The solid tumor cell lines, including the PLC, BEL-7404 and Huh7 liver cancer cell lines and the H1299 lung cancer cell line, were further analyzed using PPA-based lectin blot analysis. As shown in Fig. 3, these four cell lines exhibited different glycosylation patterns and the bands were primarily between 55 and 100 kDa. These findings indicate that PPA-based lectin blot analysis could be used to distinguish between glycosylation patterns in various cancer cell types, including types of blood cancer and solid tumors.

## Discussion

Due to their oligosaccharide specificity, lectins have been used in a variety of biological techniques, including lectin array, lectin blot analysis and lectin-based chromatography (17). In previous studies, lectin blot analysis has been used to detect the glycosylation of proteins, including an *Aspergillus oryzae* lectin blot for probing N-glycans containing core fucose (18), an *Aleuria aurantia* lectin blot for detecting the fucosylation of β-haptoglobin and for providing biomarkers for colon cancer (19), and a phytohemagglutinin lectin blot for analyzing changes in N-glycan patterns for integrins, which is involved in epithelial to mesenchymal transition of epithelial cells (20). All these lectin blot analysis techniques were mainly used for detecting the glycosylation of specific molecules. However, unlike the lectin blots used in these previous studies, the PPA-based lectin blot analysis technique used in the present study successfully recognized typical glycosylation patterns in various cancer cell lines and the glycofiles were found to differ significantly among these cell lines. The glycofiles detected using PPA-based lectin blot analysis may provide a ‘glycosylation fingerprint’ for a variety of cancer cells and may be valuable for cancer prognosis and diagnosis.

## References

[1] Mattaini KR, Vander Heiden MG (2012). Cancer. Glycosylation to adapt to stress. Science.

[2] Kim YS, Ahn YH, Song KJ (2012). Overexpression and β-1,6-N-acetylglucosaminylation-initiated aberrant glycosylation of TIMP-1: a ‘double whammy’ strategy in colon cancer progression. J Biol Chem.

[3] Saeland E, Belo AI, Mongera S (2012). Differential glycosylation of MUC1 and CEACAM5 between normal mucosa and tumour tissue of colon cancer patients. Int J Cancer.

[4] Sharon N, Lis H (1989). Lectins as cell recognition molecules. Science.

[5] Fry SA, Afrough B, Lomax-Browne HJ (2011). Lectin microarray profiling of metastatic breast cancers. Glycobiology.

[6] Drake PM, Schilling B, Niles RK (2012). Lectin chromatography/mass spectrometry discovery workflow identifies putative biomarkers of aggressive breast cancers. J Proteome Res.

[7] Wu J, Xie X, Liu Y (2012). Identification and confirmation of differentially expressed fucosylated glycoproteins in the serum of ovarian cancer patients using a lectin array and LC-MS/MS. J Proteome Res.

[8] Li C, Simeone DM, Brenner DE (2009). Pancreatic cancer serum detection using a lectin/glyco-antibody array method. J Proteome Res.

[9] Batabyal SK, Majhi R, Basu PS (2009). Clinical utility of the interaction between lectin and serum prostate specific antigen in prostate cancer. Neoplasma.

[10] Ahn YH, Shin PM, Oh NR (2012). A lectin-coupled, targeted proteomic mass spectrometry (MRM MS) platform for identification of multiple liver cancer biomarkers in human plasma. J Proteomics.

[11] Li GC, Li N, Zhang YH (2009). Mannose-exposing myeloid leukemia cells detected by the sCAR-PPA fusion protein. Int J Hematol.

[12] Chen K, Yang X, Wu L (2013). *Pinellia pedatisecta* agglutinin targets drug resistant K562/ADR leukemia cells through binding with sarcolemmal membrane associated protein and enhancing macrophage phagocytosis. PLoS One.

[13] Lu Q, Li N, Luo J (2012). *Pinellia pedatisecta* agglutinin interacts with the methylosome and induces cancer cell death. Oncogenesis.

[14] Cao J, Guo S, Arai K, Lo EH, Ning M (2013). Studying extracellular signaling utilizing a glycoproteomic approach: lectin blot surveys, a first and important step. Methods Mol Biol.

[15] Qiu Y, Patwa TH, Xu L (2008). Plasma glycoprotein profiling for colorectal cancer biomarker identification by lectin glycoarray and lectin blot. J Proteome Res.

[16] Ferguson RE, Jackson DH, Hutson R (2005). Detection of glycosylation changes in serum and tissue proteins in cancer by lectin blotting. Adv Exp Med Biol.

[17] Clark D, Mao L (2012). Cancer biomarker discovery: lectin-based strategies targeting glycoproteins. Dis Markers.

[18] Mun JY, Lee KJ, Kim YJ (2012). Development of fluorescent probes for the detection of fucosylated N-glycans using an *Aspergillus oryzae* lectin. Appl Microbiol Biotechnol.

[19] Park SY, Lee SH, Kawasaki N (2012). α1-3/4 fucosylation at Asn 241 of β-haptoglobin is a novel marker for colon cancer: a combinatorial approach for development of glycan biomarkers. Int J Cancer.

[20] Xu Q, Isaji T, Lu Y (2012). Roles of N-acetylglucosaminyltransferase III in epithelial-to-mesenchymal transition induced by transforming growth factor β1 (TGF-β1) in epithelial cell lines. J Biol Chem.

